# Integrating computational engines to identify *TSPAN6* as a migrasome-associated target for immunotherapy sensitization

**DOI:** 10.3389/fimmu.2026.1782717

**Published:** 2026-02-17

**Authors:** Liwen Fang, Hui Pan, Yihao Zhu

**Affiliations:** 1 Department of Clinical Laboratory, Changhai Hospital, Naval Medical University, Shanghai, China; 2 Clinical Cancer Institute, Center for Translational Medicine, Naval Medical University, Shanghai, China

**Keywords:** bioinformatics, immunotherapy, microenvironment, migrasome, multi-omics

## Abstract

Migrasomes, recently discovered extracellular organelles, are implicated in cancer progression and immune regulation. Nevertheless, their roles in cancer immunotherapy resistance remain poorly understood. To address this gap, we integrated cutting-edge computational engines to identify migrasome-associated targets modulating cancer immunotherapy. Using the Cancer Immunology Data Engine (CIDE) covering 5,957 patients across 17 tumor types, TSPAN6 was identified as significantly associated with adverse immunotherapy outcomes. Pan-cancer validation across the TCGA, ICGC, and CPTAC cohorts confirmed that elevated TSPAN6 expression significantly correlates with adverse prognosis. Using the pan-cancer atlas of over 4.4 million cells, we revealed the specific expression of TSPAN6 in malignant cells. Additionally, TSPAN6-high malignant cells significantly up-regulate immune checkpoint genes including CD274, NECTIN2, and LGALS9, thereby enhancing immunosuppressive interactions with exhausted T cells. Genetic ablation of TSPAN6 in co-culture models enhanced anti-tumor immunity, functionally validating this mechanism. Spatial transcriptomics further demonstrated TSPAN6 enrichment in tumor cores and its significant downregulation in immunotherapy responders compared to non-responders. In our validation cohorts, paired serum samples from 44 cancer patients showed significantly decreased TSPAN6 levels following immunotherapy. To overcome TSPAN6-mediated resistance, we computationally screened 1,615 FDA-approved compounds for inhibiting TSPAN6. Among these drugs, mitoxantrone demonstrated high-affinity binding to TSPAN6 through hydrogen bonding and hydrophobic interactions with TSPAN6. Collectively, our findings establish TSPAN6 as a migrasome-related regulator driving adverse immunotherapy outcomes and responses. Targeting TSPAN6, potentially with mitoxantrone, presents a potential strategy to enhance immunotherapy efficacy.

## 1 Introduction

Immunotherapy has revolutionized oncology by achieving durable responses across diverse malignancies, yet primary and acquired resistance persist remain a major therapeutic barrier for a significant proportion of patients (1). While immune checkpoint blockade efficacy depends on cytotoxic T-cell infiltration, tumor-intrinsic mechanisms driving immune evasion remain incompletely understood. Emerging evidence suggests that migrasomes, newly discovered organelles that were initially identified in migrating cells by Yu’s research group (2, 3), may serve as key mediators of tumor progression (4). Migrasomes are extracellular, membrane-bound vesicular structures of different sizes, typically positioned at the tips or intersections of retraction fibers (5). The biogenesis of migrasomes is tightly coupled to cell migration, proceeding through the nucleation, maturation, and expansion phases (6). During migration, retraction fibers gradually accumulate in cytoskeletal components and specific membrane proteins (such as tetraspanins) are extruded from the trailing edges of cells (5). Membrane swelling at fiber breakpoints or termini ultimately leads to the formation of migrasomes. These structures can release their contents, which can be taken up by surrounding cells, thereby facilitating various biological processes. Recent studies reveal that migrasomes participate in processes spanning cellular homeostasis to intercellular communication, such as mitochondrial quality control (7), autophagosome/lysosome fusion (8), angiogenesis, and morphogenesis (9).

As studies on migrasomes continue to grow, their involvement in cancer is becoming increasingly elucidated. Given the rapid growth and metabolic characteristics of cancer cells, they frequently form migrasomes during proliferation or migration (5). These dynamic vesicular structures can exacerbate cancer aggressiveness through angiogenesis or extracellular matrix remodeling (3), promote immunosuppression by transporting molecules like programmed cell death ligand 1 (PD-L1) or vascular endothelial growth factor (VEGF) (4, 10), and establish pro-tumorigenic niches through chemokine secretion (5, 11). Recent studies also indicate strong correlations between higher expression of migrasome-related genes and adverse outcomes in various cancers (12, 13), suggesting migrasomes as potential immunotherapeutic targets. Although these studies provide insight into the potential involvement of migrasomes in cancer immunotherapy, their roles in cancer immunotherapy resistance remain largely unexplored, and no migrasome-targeted therapies exist in current clinical practice. Therefore, further comprehensive and direct studies are needed.

**Figure 1:**
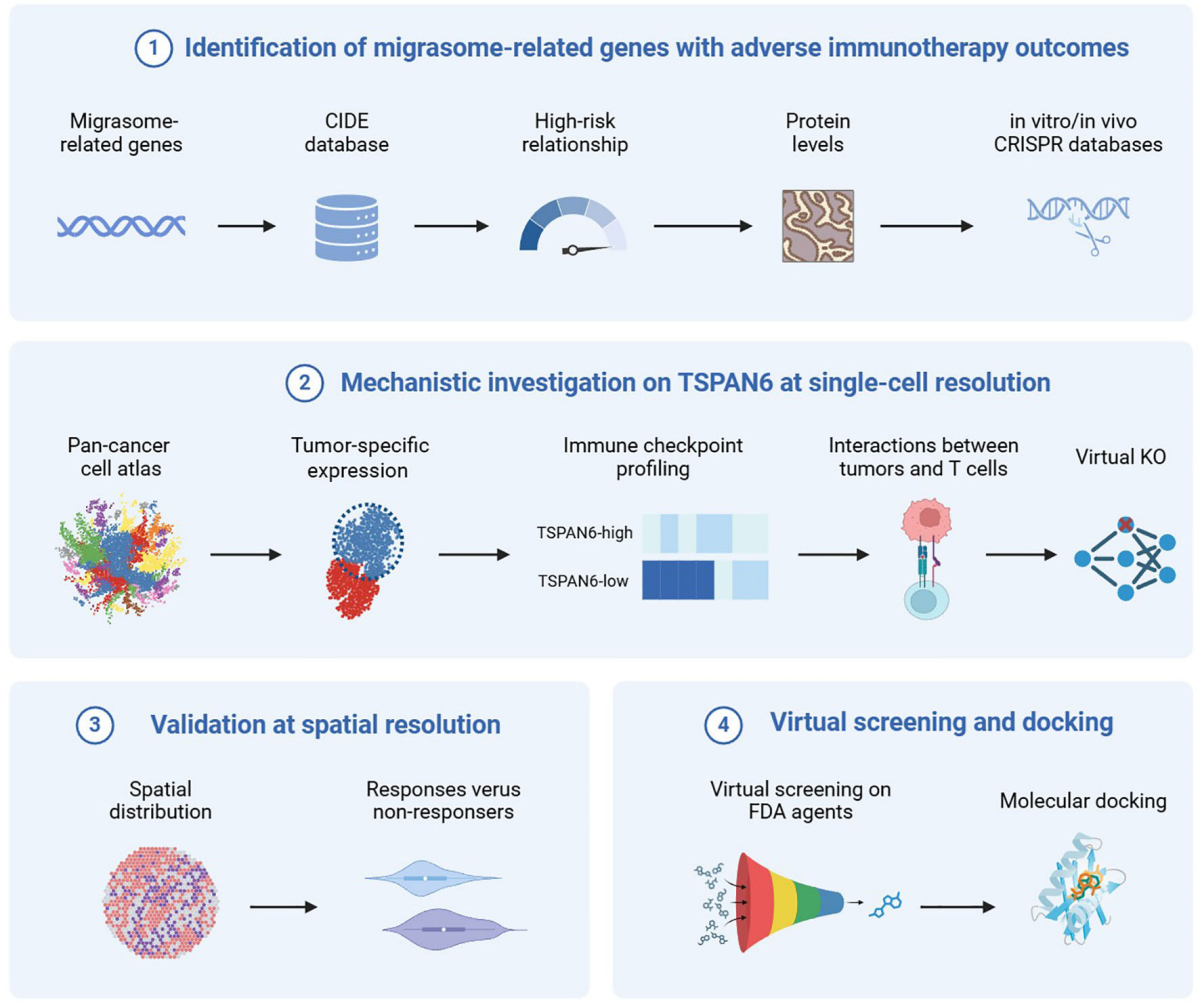
Our study comprised four parts, including (1) identification of migrasome-related genes associated with adverse immunotherapy outcomes (2); mechanistic investigation of TSPAN6 at single-cell resolution (3); validation of TSPAN6 at spatial resolution, and (4) virtual screening of TSPAN6 inhibitors. **Figure 1** was created with Biorender.com with permission.

Advances in large-scale immunotherapy data engines and high-resolution transcriptomics enable comprehensive dissection of cancer resistance mechanisms (14). The tumor immune microenvironment (TIME) is a complex ecosystem where cancer-immune cell interactions are found to influence response to therapies (15). Emerging evidence indicates that PD-L1 concentrates at the rear of migrating cancer cells (16), facilitating both sustained cellular migration and migrasome formation. Notably, migrasomes produced by pancreatic cancer cells are enriched with chemokines and cytokines, which promote an immunosuppressive microenvironment by recruiting and reprogramming macrophages (17). Given the role of migrasomes in information transmission, we speculated that migrasome-mediated cell communication may play a critical role in cancer immunity. Accordingly, we hypothesize that migrasome-derived factors sustain immunotherapy resistance by remodeling the TIME. To validate this premise, we integrated cutting-edge computational engines to identify migrasome-associated target for immunotherapy sensitization. This includes large-scale target prediction, mechanistic investigation at both single-cell and spatial resolutions, and virtual inhibitor screening, as shown in **Figure 1**. Through this integrated approach, we discovered Tetraspanin 6 (TSPAN6) as a migrasome-associated target linked to adverse immunotherapy response. Our findings suggest TSPAN6 contributes to immunotherapy resistance through three mechanisms: malignant cell-specific enrichment across a wide spectrum of solid tumors; coordinated exclusion of CD8^+^ T cells with concurrent immune checkpoint upregulation; and spatial accumulation in tumor cores of non-responders. In paired samples from our in-house cohorts, serum TSPAN6 levels decreased significantly post-immunotherapy, supporting its utility as a response biomarker. Virtual screening identified mitoxantrone as a TSPAN6 inhibitor showing high affinity, revealing direct pharmacologic targeting potential. Collectively, our work establishes migrasomes as immunotherapy resistance modulators and proposes TSPAN6-targeted strategies to overcome it.

## 2 Materials and methods

### 2.1 Cancer immunology data engine

Cancer Immunology Data Engine (CIDE) (https://cide.ccr.cancer.gov/) integrates 90 omics datasets encompassing 5,957 patients with immunotherapy outcomes from 17 solid tumors types (18). The detailed information on these datasets can be found in **Supplementary Table 1**. Outcome metrics include response binary (response versus non-response), Response Evaluation Criteria in Solid Tumors (RECIST), overall survival (OS), progression-free survival (PFS), and relapse-free survival (RFS). The association between gene expression and immunotherapy outcomes was evaluated using risk scores according to different types of outcomes. Cox proportional hazard (PH) regression, ordinary least squares (OLS), and two-sided Wilcoxon rank-sum test were implemented for survival outcomes (OS, PFS, and RFS), RECIST outcomes, and response binary outcomes, respectively. A curated list of 35 migrasome-related genes was retrieved from our previous study (19). This covers membrane markers (TSPAN1, TSPAN2, TSPAN3, TSPAN4, TSPAN5, TSPAN6, TSPAN7, TSPAN9, TSPAN13, TSPAN18, TSPAN25, TSPAN26, TSPAN27, ITGA1, ITGA3, ITGA5, ITGB1), protein markers (NDST1, EOGT, PIGK, CPQ), regulator molecules (ROCK1, TGFB2, IL1B, PDGFD, CXCL12, WNT8A, WNT11, MYDGF, BMP1, BMP7, CXCL18, WNT5B, LEFTY1, BMP2), comprehensively contributes to migrasome biogenesis. For migrasome-related covered in CIDE, their risk scores were computed, and the P-values of risk scores across cohorts were calculated using the two-sided Wilcoxon signed-rank test. The negative and positive risk score represent a favorable and adverse outcome, respectively. Genes were ranked by the absolute median risk score, with the top 2 (absolute median risk score > 0.5) selected.

### 2.2 TIMER3

TIMER3 (https://compbio.cn/timer3/) includes TCGA and large cancer cohorts (20). We analyzed the differential expressions of TSPAN6 and BMP1 across the tumor and normal tissues across the TCGA cohort. Deconvolution methods spanning 15 state-of-the-art algorithms were systemically used to estimate the infiltration levels of immune cells in tumor tissues. To ensure the robust of immune infiltration estimation, the correlation results were subjected to tumor purity adjustment. The relationships between infiltration levels of immune cells (including CD8^+^ T cells, CD4^+^ T cells, macrophages, myeloid-derived suppressor cells, dendritic cells, and NK cells) and TSPAN6 expression in tumor tissues were investigated using the partial Spearman’s correlation approach. The relationships between expression patterns of immune checkpoint genes and TSPAN6 in tumor tissues was also explored using the Spearman’s correlation approach.

### 2.3 Human protein Atlas

The protein expressions of TSPAN6 and BMP1 across different types of cancers were explored using the human protein atlas (HPA). The immunohistochemistry (IHC) staining images mapping the protein expressions of TSPAN6 in liver cancer, lung cancer, and prostate cancer were acquired from the HPA (https://www.proteinatlas.org/).

### 2.4 Immune-related CRISPR screen analyzer of functional targets

ICRAFT (https://icraft.pku-genomics.org/#/homepage) contains a vast array of genome-scale CRISPR screens focusing on the immune-modulatory effects of genes (21). We explored the TSPAN6 and BMP1 knockout (KO) effects on cancer cells, co-cultured with immune cells (NK cells, CD8^+^ T cells, and CAR-T). The statistical evaluation was performed using permutation of ranks and adjusted by false discovery rate (FDR). Negative z-score normalized Log_2_FC values indicate that gene KO will increase sensitivity of cancer cells to immune cell-mediated killing, while positive values indicate that it will decrease the sensitivity.

### 2.5 Tabula of the tumor immune microenvironment

Tabula of the tumor immune microenvironment (TabulaTIME) (http://wanglab-compbio.cn/TabulaTIME/) integrates 103 tumor single-cell RNA-seq (scRNA-seq) studies, yielding a pan-cancer resource of 4,483,367 cells from 746 patients across 36 types of solid tumor (22). This framework encompasses data processing, MetaCell identification, lineage integration, and subtype characterization. Source code and merged data are available at the TabulaTIME server (http://wanglab-compbio.cn/TabulaTIME/). We obtained a pan-cancer TME atlas comprising 140,072 MetaCells annotated with 15 cell types and 56 cell subtypes. The cell types include CD8^+^ T cells, conventional CD4^+^ T cells, regulatory T cells (Treg), NK cells, proliferating T cells (Tprolif), B, plasma cells, monotypes/macrophages (Mono/macro), dendritic cells (DC), mast cells, endothelial cells, fibroblasts, myofibroblasts, epithelial cells, and malignant cells. The atlas was imported and visualized using the R packages Seurat (23) and plot1cell (24). Expression of TSPAN6 was mapped using the R package Nebulosa (25). Malignant cells were divided into TSPAN6-high and TSPAN6-low groups according to the median expression value of TSPAN6. Expression levels of CD274, NECTIN2, and LGALS9 between TSPAN6-high and TSPAN6-low malignant cells were assessed using the two-sided Wilcoxon signed-rank test.

### 2.6 CellChat

The R package CellChat (26) was employed to quantify cell-cell communication probabilities between malignant cells and exhaustion T cells (Tex) based on ligand-receptor expression from scRNA-seq data. Significance of ligand-receptor interactions between two cell sub-populations was determined by permutation testing.

### 2.7 scTenifoldKnk

Virtual knockout of TSPAN6 in malignant cells using the R package scTenifoldKnk (27). The expression profiling of top 5000 highly variable genes from malignant cells was extracted for constructing gene regulatory network. TSPAN6 was then virtually knockout. The parameters were set as: qc_mtThreshold =0.1, qc_minLSize =500, nc_nNet =10, nc_nCells =200, nc_nComp =3. Differentially regulated genes were then identified before and after virtual TSPAN6 knockout according to the Z-score and FDR, and KEGG enrichment analysis was conducted using the R package clusterProfiler.

### 2.8 SpaCET

Spatial Transcriptomic (ST) data from 7 hepatocellular carcinoma patients with anti-PD-1 treatment, including 4 responders and 3 non-responders, was retrieved from the Gene Expression Omnibus (GEO) database under accession GSE238264 (28). The R package Spatial Cellular Estimator for Tumors (SpaCET) was used to infer cell identities (29). Spots with < 100 genes detected were excluded. Fraction of malignant, immune, and stromal lineages was deconvolved using the reference single-cell transcriptomic of hepatocellular carcinoma. We then mapped the spatial composition of TSPAN6 in malignant cells. The differential expression levels of TSPAN6 between responders and non-responders were assessed by the two-sided Wilcoxon signed-rank test.

### 2.9 Patient population and blood sample collection

A total of 44 tumor patients who were diagnosed pathologically at the First Affiliated Hospital of Naval Medical University were included in this study. The blood samples used were the remaining discarded samples from the routine clinical tests of patients. The information of enrolled patients, including age, gender, tumor type, stage, immunotherapy regimen, treatment initiation time, collection time after treatment and duration (days), was extracted and recorded from the electronic medical record system, as shown in the **Supplementary Table 2**. Blood samples were collected following the standard venous blood collection procedure. Anticoagulant blood collection tubes containing EDTA were used, and approximately 5 mL of peripheral venous blood was collected from each patient. After blood collection, the samples were kept at room temperature (25 °C) for no more than 30 minutes, and then centrifuged to obtain plasma. Subsequently, plasma samples without hemolysis, lipemia, or jaundice were selected for study, and all samples were completely anonymized. This study was reviewed and approved by the Ethics Committee of the First Affiliated Hospital of Naval Medical University (CHEC2025-198.).

### 2.10 In house validation of TSPAN6

Blood samples were centrifuged within 30 minutes after collection. Centrifuge tubes at 1500g at room temperature for 10 minutes. After centrifugation, carefully aspirate the upper-layer plasma, avoiding the middle white blood cell layer and lower red blood cell layer. Immediately test the plasma or store at -80 °C until ELISA initiation.

TSPAN6 was detected using the Thermo Fisher Varioskan LUX microplate reader and double-antibody sandwich ELISA (Cusabio), strictly following kit instructions. Standards and samples were added to the wells of a 96-well plate. The plate was incubated at 37 °C for 2 hours. Subsequently, the plate was washed 3 times using a washer. Biotinylated detection antibody was added to the wells and incubated at 37 °C for 1 hour, followed by washing. HRP-streptavidin was added and incubated at 37 °C in the dark for 1 hour, followed by another wash. TMB substrate was added and allowed to react at 37 °C in the dark for 15 to 30 minutes. Finally, the stop solution was added, and the absorbance (OD) values of each well were measured at a primary wavelength of 450 nm using the microplate reader. TSPAN6 concentrations (pg/mL) were calculated using the standard curve. All samples were tested in triplicate to ensure repeatability and reliability. The differences in TSPAN6 concentration between pre-immunotherapy and post-immunotherapy were assessed using the paired Wilcoxon signed-rank test.

### 2.11 Virtual screening

A total of 1615 FDA-approved drugs were downloaded from the ZINC database (https://zinc.docking.org/) (30). The protein structure of TSPAN6 was predicted using the AlphaFold3 tool (https://alphafoldserver.com/). BatchVinaGUI was used to perform virtual screening to batch docking the 1615 FDA-approved drugs and TSPAN6 (31). The top 5 drugs including dantrolene, mitoxantrone, nitisinone, rabeprazole, and gentamicin were selected based on high affinity. Among these five drugs, mitoxantrone was selected to dock with TSPAN6. the binding site between TSPAN6 and mitoxantrone was implemented using the CB-DOCK2 (https://cadd.labshare.cn/cb-dock2/) (32).

## 3 Results

### 3.1 Integrating computational engines identified TSPAN6 as a migrasome-related target associated with adverse immunotherapy outcomes

**Figure 2:**
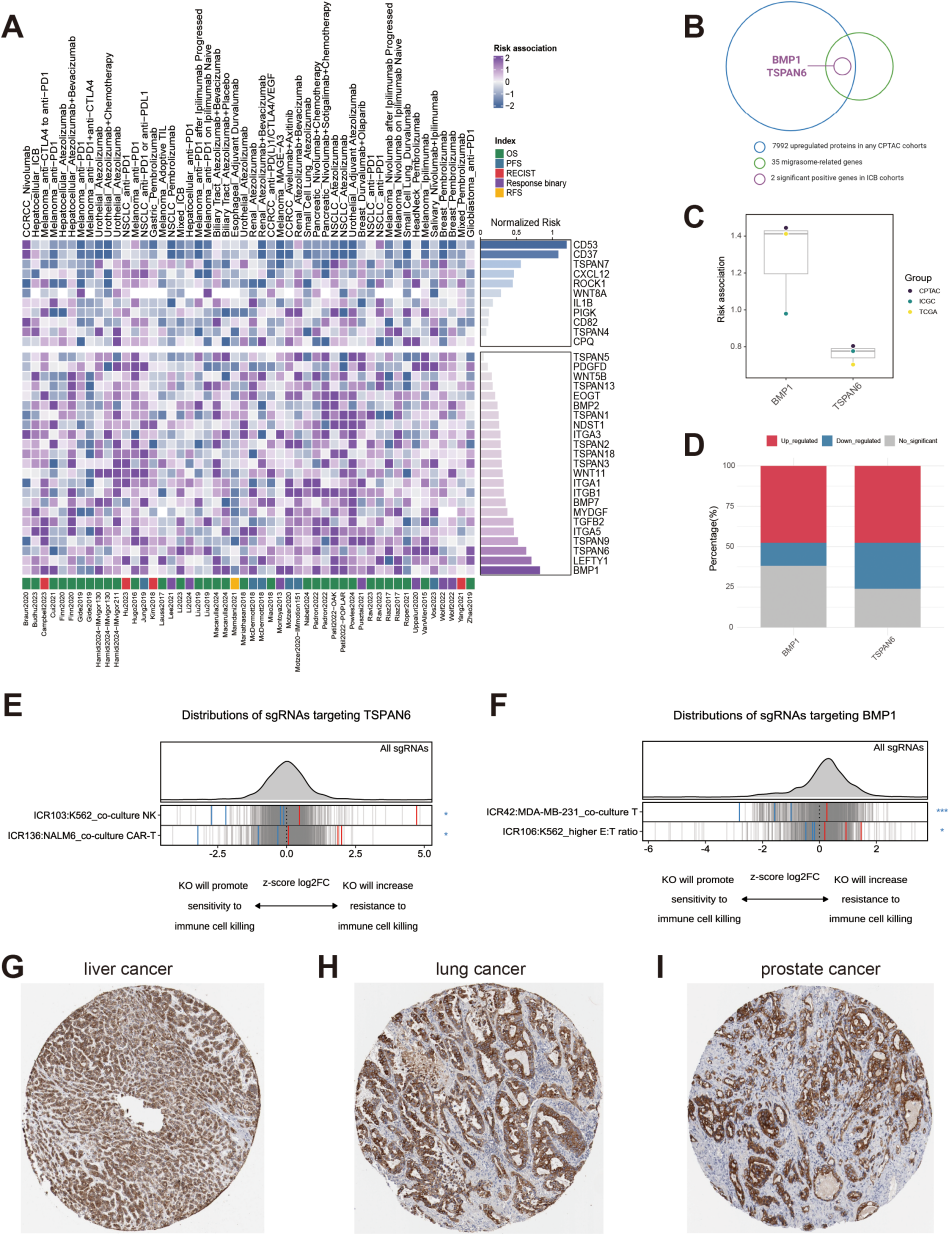
Identification of TSPAN6 as migrasome-related genes with significant adverse immunotherapy outcomes. **(A)** Prioritization of 35 migrasome-related genes using CIDE. **(B)** Identification of BMP1 and TSPAN6 as migrasome-related genes with significant adverse immunotherapy outcomes. **(C)** The risk association of BMP1 and TSPAN6 within the TCGA, ICGC, and GEO datasets. **(D)** The proportion of up-regulations, down-regulations, and non-significant regulations of BMP1 and TSPAN6 in the TCGA datasets. **(E)** Distribution of Z-score normalized log2FC values of sgRNAs targeting TSPAN6 in tumor cells, co-cultured with immune cells. **(F)** Distribution of Z-score normalized log2FC values of sgRNAs targeting BMP1 in tumor cells, co-cultured with immune cells. **(G)** The protein expression of TSPAN6 elevated in liver cancer. **(H)** The protein expression of TSPAN6 elevated in lung cancer. **(I)** The protein expression of TSPAN6 elevated in prostate cancer.

We analyzed data from the Cancer Immunology Data Engine (CIDE), a newly released resource that aggregates 90 omics datasets from 5,957 patients across 17 solid tumor types, all with associated immunotherapy outcomes (7). For 35 migrasome-related genes covered in CIDE, we calculated risk scores to quantify the association between their expression levels and immunotherapy outcomes. As depicted in **Figure 2A**, these 35 migrasome-related genes were classified based on median risk scores, with scores > 0.5 indicating adverse outcomes and scores < 0.5 indicating favorable outcomes. To identify migrasome-associated targets for immunotherapy sensitization, we overlapped 7,922 proteins upregulated in any CPTAC dataset with migrasome-related genes significantly associated with adverse outcomes (risk score > 0.5). BMP1 and TSPAN6 were thus identified, as shown in **Figure 2B**. We then investigated the prognostic association of BMP1 and TSPAN6 across the TCGA, ICGC, and CPTAC databases (**Figure 2C**). Consistently, higher expression of BPM1 and TSPAN6 was associated with adverse prognosis (risk > 0.5). Furthermore, we noted upregulation of TSPAN6 in approximately half of the tumors within the TCGA database (**Figure 2D**; **Supplementary Figure 1**). Using the ICRAFT database, we investigated the knockout effect of TSPAN6 and BMP1 in malignant cells co-cultured with immune cells. As demonstrated in **Figure 2E** and **Figure 2F**, the negative Z-score normalized log_2_FC values observed for TSPAN6 and BMP1 in different malignant cells upon co-culture with immune cells suggested increased sensitivity of cancer cells to immune cell-mediated killing. Therefore, ablation of TSPAN6 or BMP1 could increase anti-tumor immunity. We also explored the protein expression of TSPAN6 and BMP1 in cancers using the HPA database. Interestingly, moderate to strong expression of TSPAN6 was observed in the majority of malignant cells, whereas BMP1 expression was limited (**Supplementary Figure 2**). Furthermore, upregulated expression patterns of TSPAN6 were observed in liver cancer (**Figure 2G**), lung cancer (**Figure 2H**), and prostate cancer (**Figure 2I**). Altogether, these results indicate that TSPAN6 may serve as a migrasome target linked to adverse immunotherapy outcomes, suggesting its potential as a target for immunotherapy sensitization.

### 3.2 TSPAN6 correlates with tumor microenvironment at the pan-cancer level

**Figure 3:**
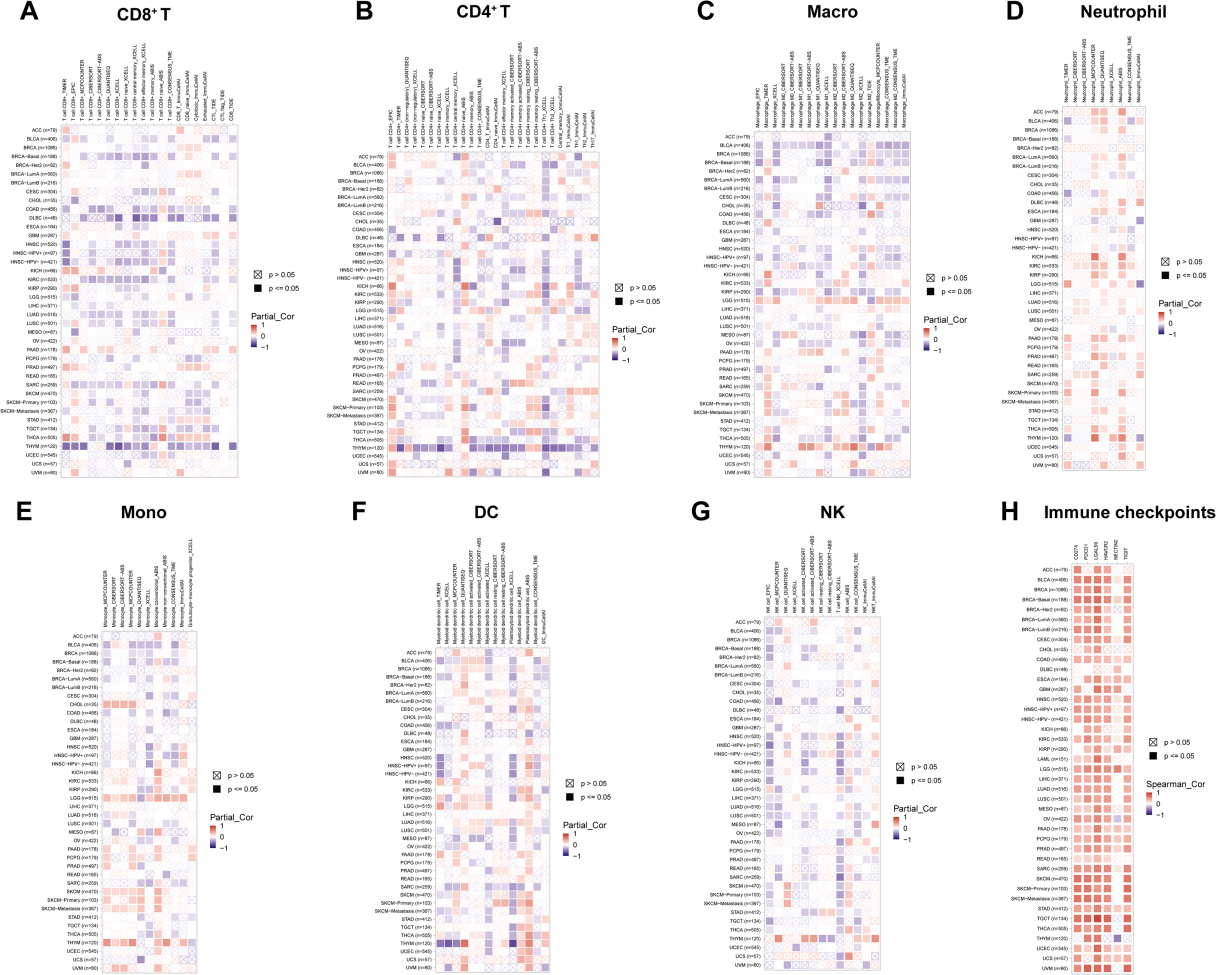
Relationship between TSPAN6 and tumor immune microenvironment at the pan-cancer level. **(A)** Correlation between TSPAN6 expression and CD8^+^ T cell infiltration levels. **(B)** Correlation between TSPAN6 expression and CD4^+^ T cell infiltration levels. **(C)** Correlation between TSPAN6 expression and macrophage infiltration levels. **(D)** Correlation between TSPAN6 expression and neutrophil infiltration levels. **(E)** Correlation between TSPAN6 expression and monocyte infiltration levels. **(F)** Correlation between TSPAN6 expression and DC infiltration levels. **(G)** Correlation between TSPAN6 expression and NK cell infiltration levels. **(H)** Correlation between TSPAN6 expression and immune checkpoint expression patterns.

Given the potential of TSPAN6 as a target for immunotherapy sensitization, we explored whether TSPAN6 expression is associated with the tumor microenvironment (TIME). Employing the TIMER3, an integrative computational tool comprising 15 state-of-the-art algorithms, we estimated the infiltration levels of immune cells within the TCGA tumors. Since immune cells are critical determinants of cancer immunotherapy response, we systematically evaluated associations between TSPAN6 expression and the infiltration levels of key immune cells (including CD8^+^ T cells, CD4^+^ T cells, macrophages, myeloid-derived suppressor cells, dendritic cells, and NK cells). Interestingly, we noted that TSPAN6 expression was significantly inversely correlated with higher infiltration levels of immune cells in most tumor types (**Figures 3A–G**). Furthermore, we explored the expression patterns of immune checkpoints (including CD274, PDCD1, LGALS9, HAVCR2, NECTIN2, TIGIT) and TSPAN6. Consistent with the TIME exclusion pattern, TSPAN6 expression was significantly positively correlated with elevated expression of these immune checkpoints (**Figure 3H**). All correlation results are summarized in **Supplementary Table 3**. Collectively, these data indicate that TSPAN6 expression negatively associates with immune infiltration and positively associates with checkpoint expression, suggesting its modulation of immune components within the TIME at a pan-cancer level.

### 3.3 Higher TSPAN6 reinforces immunotherapy resistance in malignant cells at single-cell resolution

**Figure 4:**
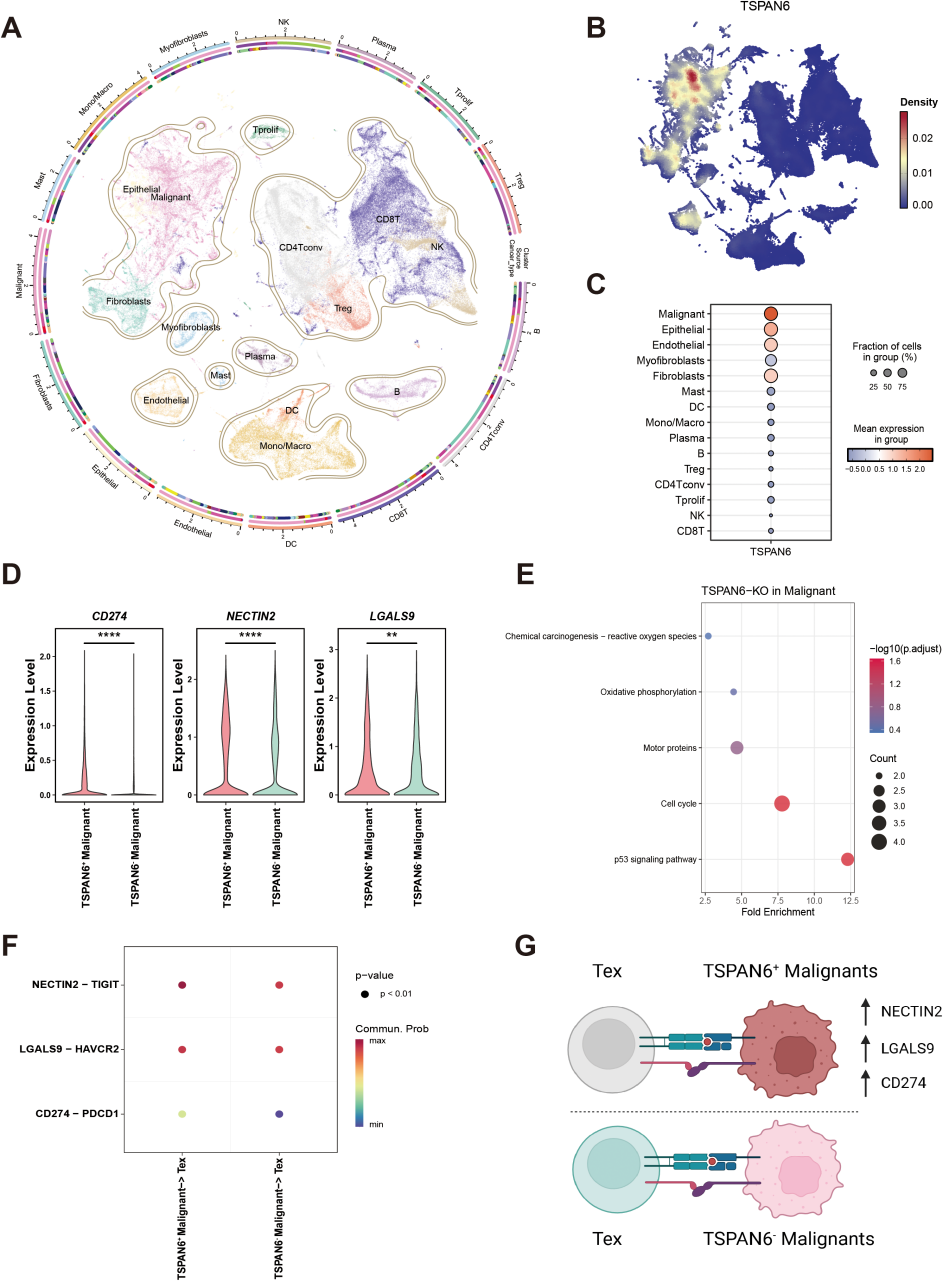
Mechanistic investigation on TSPAN6 at single-cell resolution. **(A)** Pan-cancer single-cell atlas. **(B)** Expression distribution of TSPAN6 in malignant cells. **(C)** Dot plot showing the higher expression and proportion of TSPAN6 in malignant cells. **(D)** Up-regulations of CD274, NECTIN2, and LGALS9 in TSPAN6-high malignant cells. **(E)** The perturbated pathways in malignant cells after virtual TSPAN6 knockout. **(F)** The immune checkpoint interactions (NECTIN2-TIGIT, LGALS9-HAVCR2, and CD274-PDCD1) between malignant cells and Tex cells. **(G)** Mechanistic model of immunotherapy resistance of TSPAN6+ malignant cells.

To investigate the mechanistic role of TSPAN6 in modulating the TIME, we employed the Tabula of the tumor immune microenvironment (TabulaTIME). This novel framework integrates a vast collection of scRNA-seq datasets, generating a pan-cancer atlas of 4,483,367 cells from 36 cancer types, clustered into 140,072 MetaCells representing 15 well-annotated cell types (**Figure 4A**). Mapping TSPAN6 expression density across this atlas revealed its highest enrichment in malignant cells (**Figure 4B**), with significantly elevated expression levels and cellular prevalence compared to other populations (**Figure 4C**). After stratifying malignant cells into TSPAN6-high and TSPAN6-low groups based on median expression, we assessed differential immune checkpoint expression. As shown in **Figure 4D**, key immune checkpoints (CD274, NECTIN2, and LGALS9) were significantly up-regulated in TSPAN6-high malignant cells. Intriguingly, virtual knockout (KO) of TSPAN6 in malignant cells identified differentially expressed genes (**Supplementary Table 4**; ranked by Z-score and FDR). KEGG enrichment analysis of the top 20 deregulated genes implicated perturbation of proliferation pathways (including p53 signaling and cell cycle) post-KO (**Figure 4E**). Subsequently, we used the CellChat to analyze the immune checkpoint interactions (CD274-PDCD1, LGALS9-HAVCR2, and NECTIN2-TIGIT) between malignant cells and exhausted T cells (Tex). Strikingly, the strength of all analyzed immune checkpoint interactions was enhanced between TSPAN6-high malignant cells and Tex relative to TSPAN6-low counterparts (**Figure 4F**). Collectively, these data demonstrate that elevated TSPAN6 expression reinforces immunotherapy resistance primarily by upregulating immune checkpoints (**Figure 4G**), thereby promoting T-cell evasion within the TIME.

### 3.4 TSPAN6 enriches in tumor regions and is down-regulated in immunotherapy responders at spatial resolution

**Figure 5:**
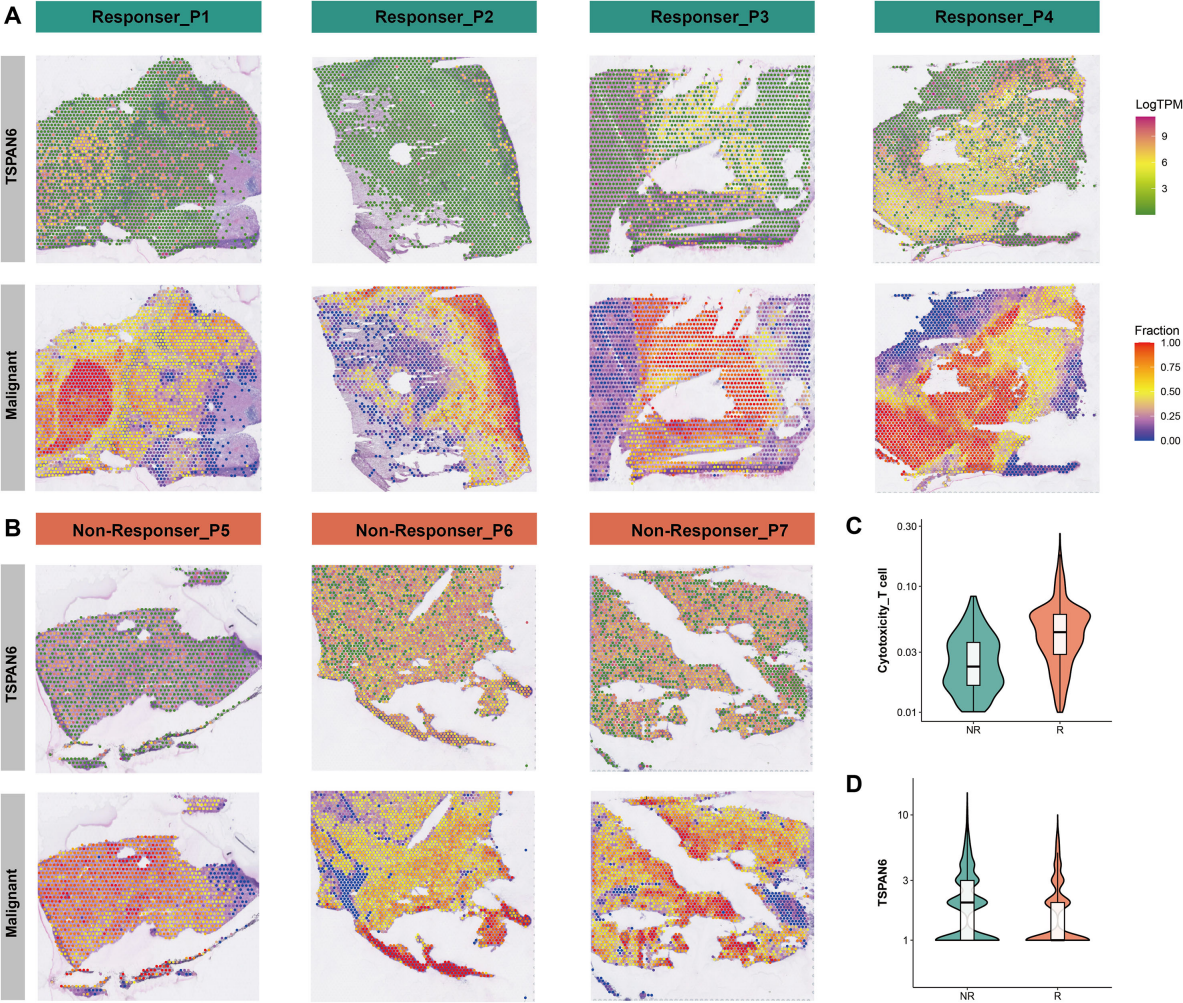
Spatial validation of TSPAN6. **(A)** The spatial expression distribution of TSPAN6 and malignant fractions in responders. **(B)** The spatial expression distribution of TSPAN6 and malignant fractions in non-responders. **(C)** Significantly higher CD8^+^ T cell scores in responders versus non-responders. **(D)** Significantly lower TSPAN6 in responders versus non-responders.

In the aforementioned analyses, we suggest the role of TSPAN6 in modulating malignant resistance at a single-cell resolution. To further investigate the spatial distribution of TSPAN6 in TIME, we analyzed the spatial transcriptomics (ST) datasets from seven hepatocellular carcinoma patients with anti-PD-1 treatment, including four responders and three non-responders. Applying the SpaCET pipeline, we evaluated the fraction of malignant cells, stromal cells, and immune cells. We also investigated the expression distribution of TSPAN6. Herein, we presented the TSPAN6 expressions and malignant fractions in each patient from responder group (**Figure 5A**) and non-responder group (**Figure 5B**). Notably, the distributions of TSPAN6 expression were mainly enriched in regions with higher malignant fractions. This data spatially validated the higher expressions of TSPAN6 in malignant cells in the scRNA-seq datasets. We further calculated CD8^+^ T cell signature scores, which were significantly elevated in responders (**Figure 5C**). Conversely, TSPAN6 expression was markedly reduced in responders relative to non-responders (**Figure 5D**). Collectively, this spatial analysis confirms TSPAN6 downregulation in immunotherapy responders and its tumor core enrichment.

### 3.5 In-house validation of TSPAN6 down-regulation after immunotherapy and screening of mitoxantrone as a potential TSPAN6 inhibitor

**Figure 6:**
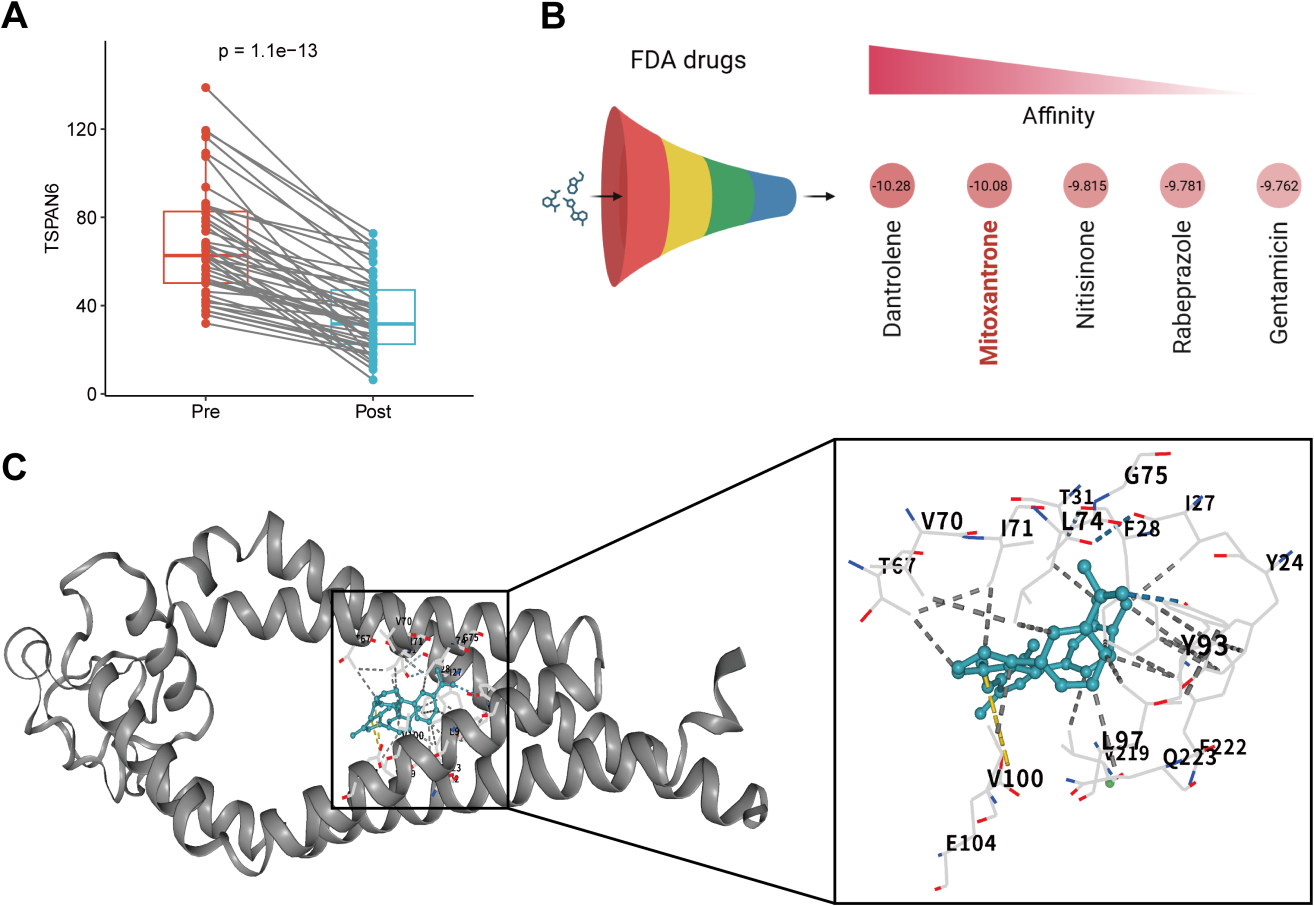
Clinical validation and virtual screening of TSPAN6 inhibitors. **(A)** Serum TSPAN6 reduction post- *versus* pre-immunotherapy (paired analysis from our in-house cohort). **(B)** Top 5 FDA-approved drug candidates ranked by binding affinity. **(C)** Left: Interaction between mitoxantrone and TSPAN6; Right: Interaction mapping (hydrogen bonds: blue dashed; hydrophobic interaction: gray dashed; ionic interaction: yellow dashed).

To validate TSPAN6 down-regulation following immunotherapy, we analyzed paired serum samples from 44 cancer patients obtained pre- and post-treatment. Analysis of our in-house cohort revealed a significant decrease in TSPAN6 levels post-immunotherapy (**Figure 6A**), supporting its potential as a non-invasive response biomarker. Given the functional role of TSPAN6 in immunotherapy resistance, we virtually screened for clinically approved inhibitors using molecular docking. After curating 1,615 FDA-approved drugs and the TSPAN6 protein structure, we computed binding affinities using the BatchVinaGUI software (**Supplementary Table 5**). The list of 1,615 FDA-approved drugs can be found in **Supplementary Table 6**. The top five compounds, including dantrolene, mitoxantrone, nitisinone, rabeprazole, and gentamicin, showed strong binding to TSPAN6 with affinities < -10 kcal/mol (**Figure 6B**). Notably, we prioritized mitoxantrone—an established topoisomerase II inhibitor with clinical antitumor activity—for detailed analysis. High-resolution docking further revealed its optimal binding pose with TSPAN6 (**Figure 6C**, left), stabilized by hydrogen bonds, hydrophobic interactions, and ionic interactions (**Figure 6C**, right).

## 4 Discussion

We identified TSPAN6 as a migrasome-associated regulator of immunotherapy resistance through integrative computational engines. This integrated approach spanning pan-cancer database mining, single-cell and spatial transcriptomics, and clinical validation demonstrates that elevated TSPAN6 expression correlates with adverse immunotherapy outcomes by potentiating an immunosuppressive tumor microenvironment. The convergence of computational, molecular, and clinical evidence positions TSPAN6 as a driver of immunotherapy resistance and a tractable therapeutic target.

Using the CIDE database, our initial computational screening of 35 migrasome-related genes prioritized candidates based on their association with adverse immunotherapy outcomes. To our knowledge, CIDE is the largest immunotherapy database, covering 8,575 tumor profiles. This represents the first attempt to identify migrasome-associated targets using CIDE. Notably, the median risk score of BMP1 (=0.827) and TSPAN6 (=0.637) significantly surpassed the threshold of 0.5, with findings corroborated by prognostic analyses in the TCGA, ICGC, and CPTAC cohorts. Importantly, functional validation revealed that genetic ablation of BMP1 or TSPAN6 enhanced tumor cell susceptibility to immune-mediated killing from co-culture models, directly linking their expression to immune evasion. While BMP1 shared similar risk associations, its limited protein expression in malignancies contrasted with the pan-cancer upregulation of TSPAN6, particularly in liver, lung, and prostate carcinomas. Accordingly, we selected TSPAN6 as a key target for immunotherapy sensitization.

TSPAN6 belongs to the tetraspanin protein family. Recent studies indicate that TSPAN6 promotes tumor progression and is associated with poor prognosis. In murine TSPAN6 knockout models, TSPAN6 deficiency inhibited TGF-α secretion through syntenin-1, attenuating EGFR signaling and exerting tumor-suppressive effects in colorectal cancer (33). Clinical studies further demonstrated that colorectal cancer patients with higher TSPAN6 expression show improved response to EGFR-targeted therapies (33), supporting its utility as a predictive biomarker. TSPAN6 also interacts with CDK5RAP3 to activate STAT3 signaling (34), transmitting activated signals via exosomes to vascular endothelial cells and inducing proliferation, migration, and angiogenesis. Higher TSPAN6 expression indicates malignant progression in glioblastoma. In gastric cancer, TSPAN6 maintains cancer stem cell properties and contributes to chemotherapy resistance through JAK1-STAT3 pathway activation (35). Accordingly, TSPAN6 represents a promising therapeutic target and prognostic marker. Elucidating its regulatory mechanisms within the tumor immune microenvironment (TIME) will advance TSPAN6-targeted therapies (36).

To investigate TSPAN6’s role in tumor microenvironment modulation, we performed pan-cancer correlation analyses. Elevated TSPAN6 levels significantly correlated with decreased immune cell infiltrations and upregulation of immune checkpoint genes (including CD274, NECTIN2, LGALS9), consistent with prior reports (37, 38). While these findings illustrate the relationships between TSPAN6 and TIME, the underlying mechanisms remained unclear. We therefore employed single-cell and spatial transcriptomics for mechanistic dissection. Using the TabulaTIME, the largest pan-cancer single-cell atlas, we demonstrated specific enrichment of TSPAN6 in malignant cells relative to other populations. Our data suggest that TSPAN6-high malignant cells drive immunosuppression through two mechanisms. First, they significantly overexpress immune checkpoint ligands (CD274, NECTIN2, LGALS9) that engaging inhibitory receptors (PDCD1, TIGIT, HAVCR2) on T cells. Second, CellChat analysis revealed intensified ligand-receptor interactions (CD274-PDCD1, NECTIN2-TIGIT, LGALS9-HAVCR2) between TSPAN6-high tumor cells and exhausted T cells, creating localized immune-privileged niches. Additionally, virtual knockout of TSPAN6 profoundly perturbed p53 signaling and cell cycle regulation, implicating additional roles in tumor growth regulation. Collectively, these findings establish TSPAN6 as a key regulator of immune checkpoints within malignant cells.

Spatial transcriptomics in HCC patients with anti-PD-1 therapy provided critical validation. TSPAN6 expression was spatially confined to regions of high malignant fractions, corroborating our single-cell observations. Additionally, responders exhibited significantly lower TSPAN6 levels with elevated CD8^+^ T cell signatures, whereas non-responders retained higher TSPAN6 expression. This finding was reinforced by serum analysis in our in-house cohort, where post-treatment showed significant TSPAN6 downregulation compared to pre-treatment. Our data support TSPAN6 as a response biomarker for immunotherapy. Given the association of elevated TSPAN6 with adverse immunotherapy outcomes, we computationally screened FDA-approved drugs for potential repurposing. Strikingly, mitoxantrone demonstrated high-affinity binding to TSPAN6 (-10.08 kcal/mol), forming stable interactions through hydrogen bonding and hydrophobic contacts. While its established antitumor mechanism involves topoisomerase II inhibition (39), our docking modeling suggests additional pharmacology through TSPAN6 inhibition. We propose that mitoxantrone may disrupt TSPAN6-mediated migrasome functions in malignant cells, potentially overcoming immunotherapy resistance.

As a central integrator of metabolism, cellular growth, and immune regulation, mammalian target of rapamycin (mTOR) has been shown to affect immune function through nuclear transcriptional programs and amino acid sensing (40). The identification of TSPAN6 as a regulator of migrasome biogenesis suggests a link between extracellular structural dynamics and intracellular metabolic-immune signaling. Recent findings underscore that amino acids act as crucial signaling molecules controlling blood glucose levels through mTOR-dependent mechanisms (41, 42). Given the role of migrasomes in releasing cellular contents, the TSPAN6-migrasome axis may modulate the release of metabolites, thereby tuning mTOR activity.

Beyond cytoplasmic sensing, our observed immune phenotypes likely involve nuclear mTOR signaling. mTOR has been shown to regulate nuclear transcriptional programs that drive cellular growth and metabolic reprogramming (40). The TSPAN6-migrasome axis potentially regulates the signaling required for these nuclear transitions, shaping the transcriptional landscape of the TIME. The physiological relevance of this crosstalk is further highlighted in chronic conditions. For instance, dysregulated mTOR signaling is a hallmark of aging and muscle-related diseases, where it compromises tissue homeostasis and regeneration (43). Collectively, these insights position the TSPAN6-migrasome axis as an upstream modulator of mTOR-mediated metabolic and immune regulation, offering a potential therapeutic target for metabolism-associated immunological disorders.

Nevertheless, several limitations should be acknowledged. While our computational analyses linked the elevated TSPAN6 expression to an immunosuppressive phenotype in malignant cells, these findings demonstrate a correlation rather than causation. Therefore, we propose the hypothesis that TSPAN6 promotes an immunosuppressive microenvironment by upregulating immune checkpoint ligands as a mechanistic model for further studies. Experimental validation is essential to confirm this causality. Furthermore, our designation of TSPAN6 as a migrasome-associated regulator is inferred from integrative computational analyses and not directly supported by experimental localization or functional assays within migrasomes.

Additionally, the virtual screening analysis is exploratory and does not substantiate the claim that mitoxantrone is a potent TSPAN6 inhibitor. Pharmacological validation of mitoxantrones in clinical cohorts and preclinical models is warranted. Moreover, our paired serum validation in 44 cancer patients provides preliminary clinical support but remains limited by sample size and tumor heterogeneity. Prospective validation in larger cohorts is required to evaluate TSPAN6 as a predictive biomarker for combination therapies.

In summary, we establish TSPAN6 as a migrasome-centered mediator of immunotherapy resistance. Multi-omics integration demonstrates its roles in prognostic stratification, TIME modulation, and therapeutic vulnerability. Targeting the TSPAN6-immune checkpoint axis may overcome immunotherapy resistance.

## Data Availability

The datasets presented in this study can be found in online repositories. The names of the repository/repositories and accession number(s) can be found in the article/**Supplementary Material**.
