# Supplementary material for: Integrating computational engines to identify TSPAN6 as a migrasome-associated target for immunotherapy sensitization
Source: Front Immunol. 2026 Feb 17;17:1782717. doi: 10.3389/fimmu.2026.1782717 (PMC12953545; doi:10.3389/fimmu.2026.1782717)
Supplement: Supplementary file 1 [file Table1.docx]

Supplementary Material

# Supplementary Figures


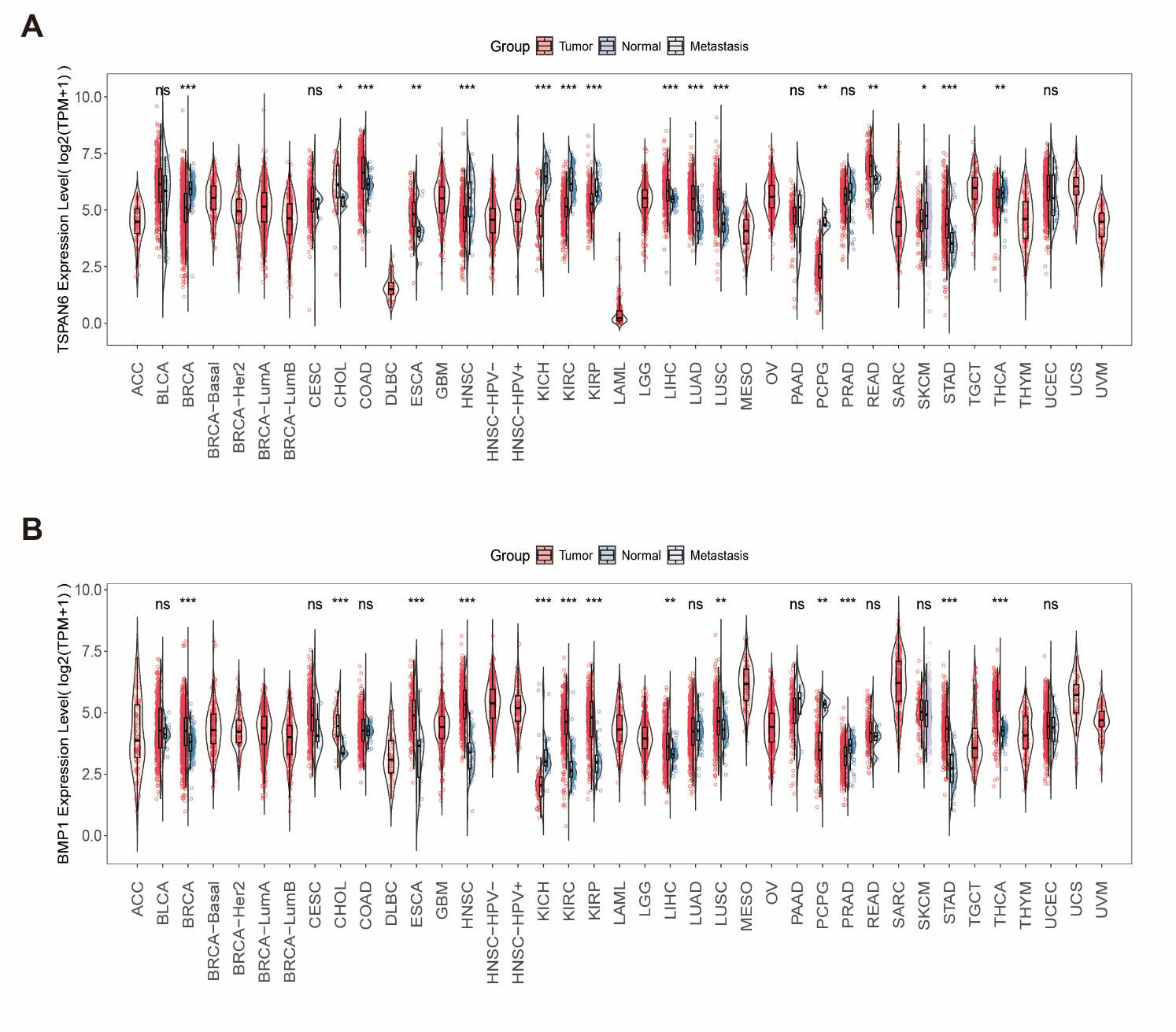


**Supplementary Figure 1.** The expression levels of TSPAN6 (A) and BMP1 (B) between normal and tumor tissues across the TCGA cohort.

**
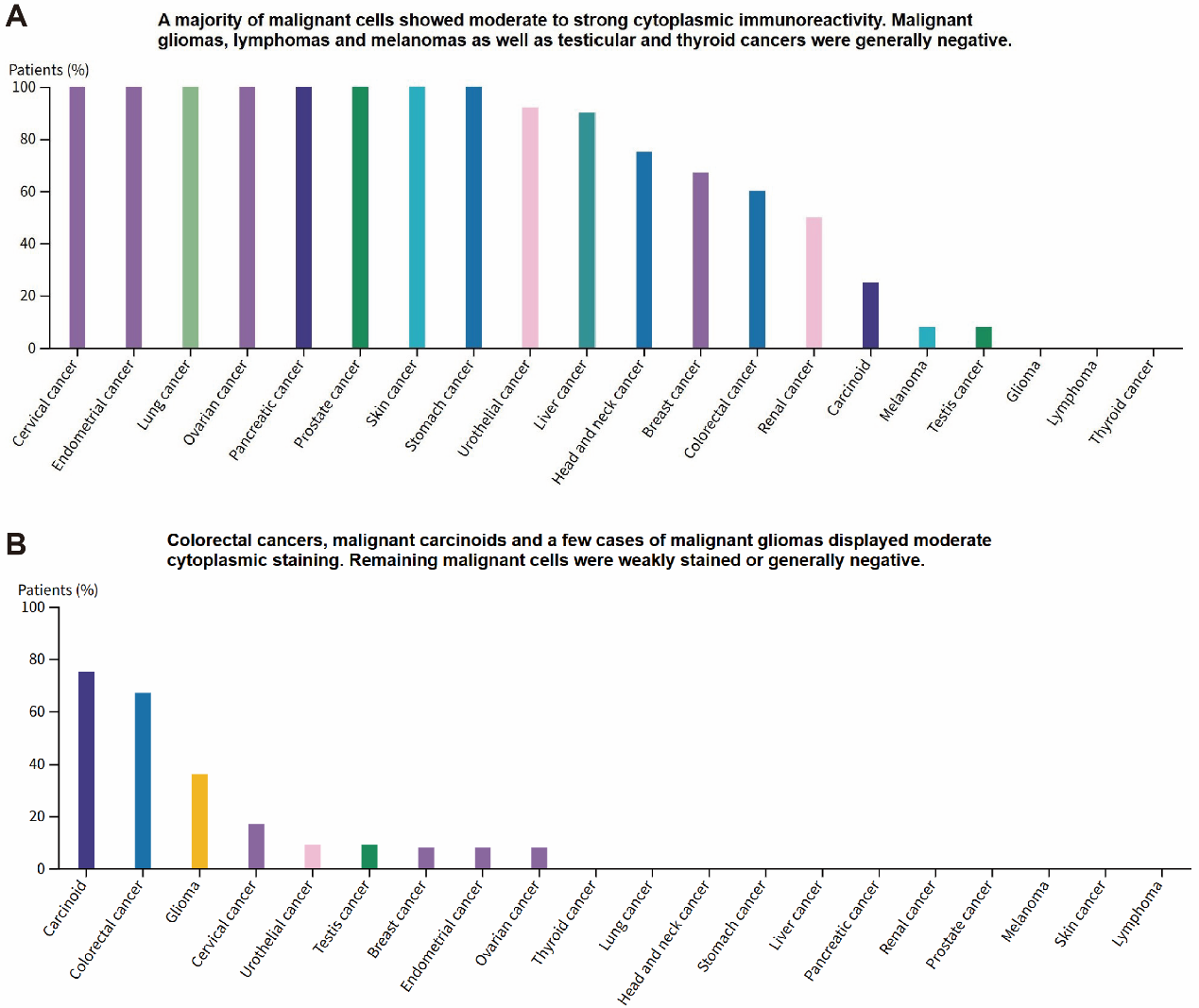
**

**Supplementary Figure 2.** The protein levels of TSPAN6 (A) and BMP1 (B) in malignances within the HPA database.

# Supplementary Tables

Please see separate excel files.

Supplementary Table 1. The detailed information on CIDE datasets.

Supplementary Table 2. The detailed information on patients in our in-house cohorts.

Supplementary Table 3. The correlation analyses between TSPAN6 and TIME.

Supplementary Table 4. The Z-score and FDR values of differential regulated genes after TSPAN6 KO.

Supplementary Table 5. The docking affinities between TSPAN6 and 1,615 FDA-approved drugs.

Supplementary Table 6. The list of 1,615 FDA-approved drugs.
